# How to make online mood-monitoring in bipolar patients a success? A qualitative exploration of requirements

**DOI:** 10.1186/s40345-021-00244-2

**Published:** 2021-12-01

**Authors:** B. Geerling, S. M. Kelders, R. W. Kupka, A. W. M. M. Stevens, E. T. Bohlmeijer

**Affiliations:** 1 Dimence Mental Health Institute, Centre for Bipolar Disorder, SCBS Bipolaire Stoonissen, Pikeursbaan 3, 7411 GT Deventer, The Netherlands; 2 Department of Psychology, Health and Technology, Centre for eHealth and Wellbeing Research, University of Twente, Enschede, The Netherlands; 3 Optentia Research Focus Area, North-West University, Vanderbijlpark, South Africa; 4 Department Psychiatry, Amsterdam Public Health Research Institute, Amsterdam UMC, Vrije Universiteit, Amsterdam, The Netherlands

**Keywords:** Bipolar disorder, Monitoring, Focus-group, Digital, Life Chart

## Abstract

**Background:**

The Life-Chart Method (LCM) is an effective self-management treatment option in bipolar disorder (BD). There is insufficient knowledge about the consumers’ needs and desires for an e-monitoring solution. The first step towards a new mood monitoring application is an extended inventory among consumers and professionals.

**Methods:**

The aim of the current study was: to identify opinions about online mood monitoring of patients with BD and professionals and to identify preferences on design, technical features and options facilitating optimal use and implementation of online mood monitoring. This study used a qualitative design with focus-groups. Participants were recruited among patients and care providers. Three focus-groups were held with eight consumers and five professionals.

**Results:**

The focus-group meetings reveal a shared consciousness of the importance of using the Life-Chart Method for online mood monitoring. There is a need for personalization, adjustability, a strict privacy concept, an adjustable graphic report, and a link to early intervention strategies in the design. Due to the fact that this is a qualitative study with a relative small number of participants, so it remains unclear whether the results are fully generalizable. We can’t rule out a selection bias.

**Conclusions:**

This study demonstrates the importance of involving stakeholders in identifying a smartphone-based mood charting applications’ requirements. Personalization, adjustability, privacy, an adjustable graphic report, and a direct link to early intervention strategies are necessary requirements for a successful design. The results of this value specification are included in the follow-up of this project.

## Background

Bipolar disorder (BD) is a severe mental illness characterized by recurrent manic, hypomanic, and depressive episodes alternating with euthymic periods. The illness often manifests in adolescence and young adulthood (Goodwin and Jamison 2012; Kupka et al. 2008). It is estimated that BD types I and II occur in 2% of the world’s population and that another 2% has a form of subthreshold BD (Geddes and Miklowitz 2013). In the Netherlands, the prevalence of BD in adults is 1.3% (de Graaf et al. 2010).

Monitoring of mood is an important element in self-management and treatment of BD. The prospective Life Chart Method (LCM) has been validated for daily mood monitoring (Denicoff et al. 2002). It provides a graphic representation of mood fluctuations above (hypo/mania) and below (depression) a euthymic baseline. Current medication, comorbid symptoms, hours of sleep, and significant life events are also reported. The primary purpose is to gain insight into the course of the illness and early signs of relapse. Daily monitoring facilitates early recognition and intervention, thereby improving personal functioning (Gershon and Eidelman 2015).

Despite these advantages, the use of the LCM can be a regular confrontation with the illness (van Bendegem et al. 2014a). Some patients use the LCM only when early signs of relapse occur and not consistently (van Bendegem et al. 2014b). Draisma et al. (2015) found that 35% of the participants had missing LCM-data during a 1-year follow-up (Draisma et al. 2015). When data are reported retrospectively, there is a considerable risk of recall bias, especially when patients complete batches of daily ratings at a single time (Stone et al. 2003; Whybrow et al. 2003).

Carefully choosing the right moment to introduce self-monitoring in the treatment and tailoring to the potential user is a critical success factor (Lysaker et al. 2014; van Bendegem et al. 2014b). In their study van Bendegem et al. (2014a) plea for further customization and personalization to optimize motivation and compliance.

Redesigning the LCM from a paper and pencil method (P&P) into a digital instrument may facilitate daily compliance (Malik et al. 2012). Over the past decades, several electronic mood monitoring systems have been developed and assessed (Faurholt-Jepsen et al. 2016; Matthews et al. 2008; Saunders et al. 2017). In some studies, digital monitoring aimed to improve compliance (e.g. by sending reminders or increasing user-friendliness) (Lieberman et al. 2010; Matthews et al. 2008). Other studies are focused on detecting differences between P&P and digital monitoring in depressive and manic symptoms (Bauer et al. 2004; Bopp et al. 2010; Depp et al. 2012; Faurholt-Jepsen et al. 2014; Schärer et al. 2002; Whybrow et al. 2003). In a systematic review, Faurholt-Jepsen et al. (2016) concluded that electronic self-monitoring of mood appears to be a reliable measure of mood in depression but not in mania (Faurholt-Jepsen et al. 2016). This finding is in contrast to the validation study of the Life Chart Method. Denicoff et al. (2000) found that the LCM ratings have a strong correlation to the Yong Mania Rating Scale and the Global Clinical Impression BD (Denicoff et al. 2000; Young et al. 1978, 2004).

A qualitative study demonstrated that e-mood monitoring could lead to a better understanding of BD, increase insights into illness, and change behaviours towards more effective self-management strategies (Saunders et al. 2017). The participants also mentioned the possibility of being too preoccupied with monitoring as a potential risk of e-mood monitoring; in addition, the participants highly appreciated the option of personalizing the monitoring system to their insights (Saunders et al. 2017). Van den Heuvel et al. (2018) found that patients had better insight into the factors that contribute to mood instability or mood episodes when they used digital monitoring (van den Heuvel et al. 2018). This underlines that electronic longitudinal mood monitoring can lead to a better impression of the course of mood fluctuations in BD (McKnight et al. 2017).

Although several studies have described patients’ experiences (including the benefits and their doubts) and measured satisfaction or user experience after development (Faurholt-Jepsen et al. 2019; Saunders et al. 2017; van den Heuvel et al. 2018), only a few studies had involved patients and professionals in (or before) the development of an online monitoring tool (e.g. Goodday et al. 2020). The current study aims to explore patients’ and professionals’ opinions about online mood monitoring (‘must-haves’ and ‘don'ts’) before developing a digital version of the widely used LCM. To the best of our knowledge, our study is the first and only study in (online) mood monitoring in bipolar disorder that involved consumers and professionals during the development process, which included the contextual inquiry, value specification, and the design of the application. The development process of interventions is described in several studies (e.g. van Gemert-Pijnen et al. 2011).

## Method

### Design

A qualitative design was used to identify and characterize the opinions about and needs (‘must-haves’ and ‘don'ts’) of relevant stakeholders (patients and professionals) of online monitoring. Three focus-group meetings (FGMs) were held. FGMs are an efficient way of gathering information on how people express their ideas on a construct (Polit and Beck 2015). FGMs are chosen as part of a qualitative design to gather participants' narratives, generate experiences and opinions, and stimulate discussion on peers’ opinions (Gray 2014; Green and Thorogood 2018). To get a broad perspective on online mood monitoring in the Netherlands, the target group was defined as patients, professionals, and developers/researchers. This approach is also known as an patient-centered digital health design (Birnbaum et al. 2015) and is an example of an initiative with enables more engaged research participation.

We designed the study to conform to holistic development principles, as stated in the CeHRes roadmap (Centre for e-Health Research and Disease management, van Gemert-Pijnen et al. 2011). This study covers the first two steps of the model, which are contextual inquiry and value specification. The CeHRes endeavours to facilitate a continuing process of evaluation and participation of all stakeholders.

Ethical approval for the study was obtained from the University of Twente (Utwente; 18067) and the scientific board of Dimence, the mental health centre where the research was conducted.

### Participants

The inclusion criteria to participate in the focus-group were: (1) patients with BD I or professionals who were treating BD or developers and researchers, (2) in possession of a smartphone, and (3) the willingness to travel to attend FGM. In the recruitment of our sample, we aimed to get a maximum variation sample to get a wide range of dimensions and a maximum variation among the participants of the FG. Therefore we use several recruitment options.

The professionals in the focus-group came from various disciplines (psychiatrists, psychiatric nurses, and psychologists). The professionals were recruited from the Dutch national knowledge institute for BD (Kenniscentrum Bipolaire Stoornissen, KenBiS), a chapter of the International Society for Bipolar Disorders (ISBD). Recruitment took place during a lecture at one of the periodic meetings of KenBiS and via leaflets.

Participating patients were recruited in an outpatient clinic and the national advocacy group (plusminus) in order to represent patients from different regions in the Netherlands and avoid potential selection bias. All the participants received treatment for BD I or II, but the treatment approaches could differ from each other due to the selection method. An announcement with a call for participation was posted in the magazine of the advocacy group. The researchers (BG and SK) were not involved in the participants’ treatment to avoid selection bias. Patients were asked if they were euthymic at the start of the FGM, although no specific questionnaire was conducted to established mood.

All participants signed an informed consent form, in which they also agreed on audio recordings during the focus-groups. Thirteen participants (eight patients and five professionals) participated in one or more FGM.

### Procedure and materials

We used the data from a previous study that aimed to gain insights from patients with BD about reasons to use, continue, or discontinue health-related apps in supporting self-management to operationalize topics for the FGM held in September and October 2018. In three semi-structured, 2-h meetings, the following topics were discussed; experiences with monitoring, technology and monitoring, the requirements of a monitoring app, and a monitoring app that can provide users with their needs. The participants were informed about the topics before the FGM. After the brief introduction of the focus-group (FG), participants were asked to write down the pros, cons and wishes on post-it memos collected on flip-charts. These statements comprised the basic assumptions used to start the discussion about the different opinions. The agreements and disagreements were summarised, and in the following FG, the members were asked again to find a preference until consensus was reached. In doing that, we gained insight into how the participants valued the current methods that give information for developing the digital version of the LC. The conversations were recorded and transcribed, and they were then followed by a consensus check. After each session, a report was made and sent to the participants for validation and comments to increase objectivity (member checking).

For the goal of mood monitoring we used the definition for the LCM as described by Denicoff et al. (2000): “*The LCM allows for the daily assessment of mood and episode severity based on the degree of mood associated functional impairment*” (Denicoff et al. 2000). The results gain insight in the course of the mood episodes and is used within the relation patient—professional to established proper treatment options.

### Data analysis

The discussion and consensus rounds were transcribed and analyzed by deductive coding.

Open coding, via ATLAS.ti, is used to process the data. Concepts of a ‘consensus document’ were discussed in the second and third meeting and finally, after the last meeting established.

## Results

### Participants

We recruited 17 participants (11 patients with BD and six mental health professionals). Three patients and one professional withdrew before the start of the study for various reasons, like not being available at the time of the FGM or having a current mood episode. One psychiatrist, one psychologist, two nurse specialists, and one psychiatric nurse represent the professionals. There was a balanced representation in the sample across age (16–24 years one person = 7.7%; 25–40 years four persons = 30.8%; 41–55 years five persons = 38.5%; and 56–70 years three persons = 23%), gender (53.8% female) and residence (rural 46.2%, urban 53.8%). In education (high school, 23%; higher professional education, 61.5%; university, 15.5%), the distribution was less balanced. Although preferences didn’t seem to differ between subgroups (e.g. age or education).

### Emerging themes FGM

The FGM were structured in five themes or categories; positive and negative experiences with monitoring, positive and negative aspects of technology and monitoring, and requirements for a monitoring app. The topics that the FG members initially brought forward (by post-it memos) are placed within these categories and summarized in Table 1. Topics that came forward from the FGM are outlined in Table 2.

**Table 1:** Overview results focus group

Topic	Code	Times initially mentioned
Positive experiences with monitoring	Provides insight	4×
	Gives direction	3×
	Insight into the course of the illness	2×
Negative experiences with monitoring	Stressful	3×
	Confrontation with the disease	3×
	Lack of privacy	2×
Positive aspects of technology and monitoring	Getting more insight	3×
	More overview options	2×
	Possibility to use notifications	4×
	Better availability	2×
	More secure	1×
Negative aspects of technology and monitoring	Need for a device	2×
	Privacy	2×
	Focus on illness	2×
	Provoke the use of the telephone	2×
Requirements for a monitoring app	**Freedom of choice** Possibility to add text/photos/videos Monitor temporary items Adding own parameters Adjustable notifications Free wording	8×
	**User-friendliness** No unnecessarily features Linking possibilities (e.g., RRP) Compatibility with other devices Notifications Clear design Easy to operate	5×
	**Trustworthiness** Clear privacy statement No hidden data extraction Reliable supplier Clear access protocol with the logging option	4×
	**Goal-setting** Personal feedback Increase of self-management Self-regulation	3×
	**Remaining** Based on the LCM	3×

**Table 2:** Additional topics

Additional topics after discussion in the FGM				
Requirements for a monitoring app				
Freedom of choice	User-friendliness	Trustworthiness	Goal-setting	Remaining
• Handle algorithms • Monitor temporary items • Choose words that apply to the user • Flexibility • Add text, photos, videos or music • Scroll function to zoom in or out on the graphic of the LC • Diary function	• Exactly meet the users’ demands • Connect with different applications • Multiple-use (phone, tablet, pc) • No daily login	• Clear privacy statement • Clear who has access to the data • Clear who can watch the LC	• Supporting in the regulation of the users • Personalized feedback	• No compulsory use • No linking with other social media

#### Experiences (positive and negative) with mood monitoring

First, we discussed the positive and negative aspects of monitoring. The Life Chart Method (LCM) was often mentioned as a monitoring tool, and some remarks refer primarily to the LCM. These valuable remarks were placed in the broad concept of mood monitoring to avoid ‘tunnel vision’ on the LCM in the FG’s early stage.

The following positive experiences were mentioned: ‘providing insight’, ‘guiding in self-management’ and ‘insight into the course of the illness over a more extended period/historical overview’. These items refer to the utility and necessity of the monitoring. Not only insight into the course of the illness was mentioned but also factors that can influence the mood (e.g., medication and sleep). Monitoring can also give direction about how to handle when early signs of relapse do occur.

*‘Provides a good insight into the course of bipolar disorder’* (participant 1).

*‘Provides insight into the combination of sleep, mood and events’* (participant 6).

*‘Provides quick insight, but must be used with the relapse prevention plan’* (participant 11).

On the opposite, participants mentioned several negative experiences or aspects of mood monitoring. Mood monitoring can be experienced as a stressful task that ‘has to be’ accomplished daily, especially in the early stage of the illness. Monitoring is also seen as a confrontation with the illness that can even induce self-stigma. When used in treatment, monitoring can also be ‘*experienced as a violation of privacy*’ since caregivers can wander through personal data.

*‘Being involved with the disease every day by the LC can eventually become compulsive’* (participant 6).

*‘It's [monitoring] not always necessary can lead to panic reactions’* (participant 4).

*‘You may also be displeased [because of the monitoring, you are too focused on the disease]’* (participant 2).

#### What could technology mean to improve monitoring?

Secondly, the use of technology as a possible solution was discussed (both potentially positive and negative). As positive aspects, the participants came forward with several expected benefits of online mood monitoring, like ‘*get more insight*’ in the monitoring and that there will be ‘*more overview options*’. The FGM expect better availability when the monitoring is always in reach (on mobile devices), the possibility to zoom in and out on the long-term course, the option to ‘*use notifications*’, and the data to be shared with their caregivers. All the aspects mentioned above contribute to better insight; the FGM didn't expect significant changes in mood monitoring base principles.

Also mentioned was; the ‘*possibility of getting feedback*’, the idea that it fits in the modern technological age, that the users have ‘*more control*’ and, because of having a personal account, ‘*better privacy*’ is guaranteed. The participants partly related their expectations to their experiences with online applications that support their P&P mood monitoring;

*‘I discovered when I did the monitoring on my desktop (in Excel, I had made a program for it) that it was easier to fill in the LC because I am sitting daily behind the desktop computer’* (participant 6).

*‘I used notifications from another app to remember me to fill in the paper and pencil LCM’*—(participant 11).

On the other hand, the participants named different possible negative aspects of online mood monitoring. The need for a device is conditional. The participants are concerned about privacy issues; access to the data has to be limited to patients, caregivers and researchers. Online mood monitoring can lead to a focus on the illness, according to the FGM.

The FG concerns are divided into two themes; technical aspect of mobile phone use and the ‘side effects’ of mobile phone use. The ‘*problems in operating the app*’ and the ‘*incompatibility with other devices*’ are mentioned as possible technical barriers. The complex operation required to use an app can reduce the motivation to use the app. The same is expected to occur when the app is not compatible with other devices (e.g., desktops). Also, ‘*too many functionalities*’ is mentioned as a possible barrier; people can get lost in all kinds of functionalities that distract them from mood monitoring’s main goal. The second theme, ‘side effects’, concerns a technical application that can ‘*provoke the use of the mobile phone*’ (too much). Even users are ‘*unwillingly switch to social media*’, especially when they are in a (hypo) manic mood episode.

*‘I don't want any interaction with other people, like on social media, because I'm afraid of what I would say when I am manic or depressed.’* (participant 4).

*‘One of the disadvantages is that you always, it could be out of power or broken (the mobile device BG); with the booklet (paper and pencil LCM), you do not have that problem.’* (participant 1).

#### (Technological) requirements

To establish perceptions of an online mood monitoring application’s essential elements, we discussed possible features and how they can tribute to compliance. The identified features can be classified into four categories*: freedom of choice, user-friendliness, trustworthiness* and *goal-setting*. The participants unanimously stressed that the LCM principles must be leading in developing the online mood monitoring application.

*Freedom of choice* seems to be one of the most important topics (8× mentioned). This topic comprises the following aspects: the application must ‘*exactly meet the users’ demands*’, the possibility to ‘*handle algorithms*’, although that may also be a bit threatening, like ‘Big Brother’. A clear wish to be able to ‘*monitor temporary items*’ and that you can ‘*choose the words that apply to you*’ in the monitoring. ‘*Flexibility*’, in addition to a fixed basis, is considered highly desirable. Also, the possibility ‘*to add text, photos, videos or even music fragments*’ was mentioned. If there are elements in the app that are personally ‘indispensable’ for you, using the app makes it more natural (for example, a link with calendar appointments).

The following items were mentioned in the FGM about *user-friendliness*: the desires to use the app on ‘*multiple applications*’ and to ‘*combine different applications*’ are also widely shared (e.g. the combination of the LC with relapse prevention plan, wellness recovery plan or mindfulness app), possibly with a link to the personal health file (PGD) or the electronic patient records (EPD).

The *trustworthiness* of the application is the third sub-category. The app must have a ‘*clear privacy statement*’ and a reliable company that markets the application. It must be clear ‘*who has access to the LC’s data*’ and ‘*who can watch the data*’*.* When others view data, one can receive messages (logging).

Finally, *goal setting* could be identified as an important topic. ‘*Goalsetting in self-management*’ and the importance of being able to ‘*regulate yourself*’ seems essential. Also, ‘*personalized feedback*’ is a motivational option to achieve monitoring goalsetting and increase compliance. In summary, the following quotes show the most important requirements as mentioned by the participants:

*‘I would like to see a kind of scroll function that you zoom in or out on the graphic of the LC; I think that would be absolutely fantastic’*—(participant 1).

*‘I would like to have a kind of diary function in the app so that I can see my appointments with the clinician, and even can monitor of the visit is worsened or improve my mood’*—Participant 2

*‘The opportunity to load up a video or audio recordings on moments that I being Euthymic so that, when I'm depressed, I can play like a kind of mantra… It could be everything; even a barking dog can you remember at good times.’*—(participant 11).

*‘Clear graph, the possibility to add notes, the option to add extra data, medication to be filled in per day, the possibility to add additional data (on or off), clarity about data exchange, applicable to multiple devices. No more functions than necessary, no chat function, no hidden data exchange.’*—(participant 6).

*‘User-friendly, using LC indicators, additional options for monitoring, sending reminders every day (to be set up), giving positive feedback.’*—(participant 8).

*‘Easy to operate, customizable, different variables, combined with a plan, in addition to having it monitored, being able to turn off notifications. No compulsory use.’*—(participant 10).

Based on the results of the FGM, the first concept of functionalities of the app was made.

#### The consensus within the FG

In the last meeting, the definitive consensus was achieved on how the monitoring application should be working. In developing an online mood-monitoring application, the LCM principles should be the priority. Adjustability and personalization are vital components to start and maintain the use of the application. Privacy must be strictly described, and the patients should own the data.

The consensus statement is shown in Table 3.

**Table 3:** Consensus monitoring

Consensus agreed on elements for online monitoring
• A basis in which all elements of the Life-Chart method are included:
◦ Functions in this part are not adjustable
◦ Notifications for monitoring are adjustable
• Graphic display of the course of mood:
◦ In weeks, months and years
◦ Possibility to adjust different parameters and to switch on/off or to combine in the graphics (for example, the parameters sleep and mood)
◦ The graphics display is adjustable (like zoom in and out)
• Additional options to adjust the app to personal circumstances and preferences:
◦ Possibility to add text/photos/music/link to the Life-Chart
◦ Possibility to add own parameters like substance use or work duration
◦ Possibility to receive personalized feedback
◦ Possibility of personalizing with photos
• Provide a link with the relapse prevention plan or with WRAP
• Adjustable notifications
• Quick to fill in for daily use
• Useable on all devices
• Guarantee for privacy:
◦ Distinctness about data storage,
◦ No access to the personal data by ICT-company or others who are not involved with the treatment of the patient
◦ The patient who is monitoring is the owner of the collected data and is the only one who can add others (like professionals) to his/her chart
• NO link with other social media to prevent uploading of personal data from the LC during an episode
• NO ‘forced’ monitoring; this will reduce the motivation for mood monitoring
• NO log in for daily use (only at the start of using the application)

## Discussion

The aim of the current study was twofold: to identify opinions about online mood monitoring of patients with BD and professionals and to identify preferences on design, technical features, and options facilitating optimal use and implementation of online mood monitoring.

Regarding the first aim, the results showed a shared consciousness of the importance of using the LCM, but only when it’s appropriate in the context of patient’s treatment and recovery process. These findings are consistent with previous studies in which both patients and professionals underscored the importance of the LCM (van Bendegem et al. 2014a, b). Lysaker et al. (2014) claims that, when not carefully integrated into the patient’s recovery process, the LCM can be contra-productive and can even lead to self-stigma and low self-esteem levels (Lysaker et al. 2007; Lysaker et al. 2014). The FG members in our study mentioned similar experiences, such as the daily confrontation with the disease that eventually can lead to self-stigma and over-focus on the disease. This underlines the necessity for flexibility. Not only flexibility in the application of the LCM (in with stage of the treatment of BD it is applied) is an important requirement for successful monitoring, but also personalization of the use of the LCM.

Regarding the second aim, our results confirm the importance of tailoring and personalization in digital monitoring tools (Saunders et al. 2017; Valenza et al. 2014). First, customization can be used in a way that patients are able to add text or photos. Also, adjustability can be integrated by using own parameters such as alcohol consumption or notifications. The FGM underlines the importance of adding (temporary) monitoring items that can influence the course of the BD, such as removal, unemployment, marriage and family extension. In BD, life events can lead to mood episodes (Koenders et al. 2014). Therefore, it can support monitoring life events more closely, even during long(er) euthymic episodes. Another aspect is the possibility to link to other self-management tools, such as a relapse prevention plan (RPP). Interventions from the RPP should appear when (mild) depression or (mild) mania is rated in the monitoring application. Such a link seems to be conditional for our participants to use online monitoring tools, which indicates the importance of personalization. In a phenomenological study of the use of the RRP, Daggenvoorde et al. (2013) found that it can be difficult for patients to put early signs of relapse into concrete prevention actions (Daggenvoorde et al. 2013). Especially in longer euthymic episodes, the RPP can be drawn to the background for some patients and can’t be applied effectively (Murray et al. 2011). If concrete early interventions, when indicated, directly appear in the monitoring application, we expect that it is easier to perform those early interventions. This pleads for integrating the RPP in the online monitoring tool to improve early interventions and improve self-control. Moreover, our results underline the importance of tailoring and personalization in a digital monitoring tool.

Besides the importance of personalization, online monitoring provides the possibility for patients and professionals to share the monitoring data at any time. Although data security has to be guaranteed, the FG members label this possibility as useful. When digital monitoring is shared with professionals, there is enhanced communication between patients and clinicians. This could lead to a better understanding of factors that influence mood (van den Heuvel et al. 2018; McKnight et al. 2017). The concerns about privacy focus on the data exchange, access to the data and in addition who can exactly can see the life-chart. Vendors should add a proper, transparent statement about data collection and view (Motti and Caine 2015). This seems conditionally for a successful implementation of an online mood monitoring system.

The results also yielded some important concerns about online monitoring. Connection with social media platforms is seen as one of the risks by the FG. The participants fear posting anything during depressed episodes and more so during (hypo)manic episodes that they will subsequently regret during euthymic episodes. Although in some papers the benefits of social media on mental health have been discussed (O’reilly et al. 2019; Välimäki et al. 2016). Naslund and Aschbrenner (2019) discovered that in a cohort of people with severe mental illness (37% with bipolar disorder), about one-third expressed concerns about privacy risks similar to our findings. The concerns are related to using social media, addressing fears of stigma, the impact on personal relationships, and facing hostility (Naslund and Aschbrenner 2019). In the FGM, the members reveal similar concerns. Therefore, it is essential to be careful in including features in applications, such as social media links, that may harm the target group.

Although health applications are widely available and a majority (90%) of patients are interested in digital interventions (Ranney et al. 2012). Only a minority of patients are actually using that kind of interventions cause of various reasons. Patients do use interventions when it fits in their needs (Lupton 2013). Like many developing groups, we held focus-group sessions to determine content and (Rosen et al. 2015). By using the model of the CeHRes, in with participation throughout the whole development process is one of the preconditions of the model, we tried to bridge the gap between the needs of end-users (patients and professionals) and the development of a new E-monitoring tool, that enables us to create a patient-centred digital health tool (Birnbaum et al. 2015; Gemert-Pijnen et al. 2011).

In sum, we argue that the results provide a number of important prerequisites for developing a mood-monitoring app that is in line with the target group’s needs. In this process, we followed the steps of the CeHRes (van Gemert-Pijnen et al. 2011). A development process using the CeHRes roadmap, can ‘clarify areas that would otherwise remain unanswered, unclear, or unknown’ (van Gemert-Pijnen et al. 2011). We believe that we have succeeded to clarify this in our study, especially possibilities for personalisation, adjustability and need for integration of self-management tools in the daily monitoring.

## Limitations

The study has some limitations. The first is a potential selection bias in the recruitment of professionals. They were voluntarily recruited in the Dutch chapter (KenBiS) of the International Society of Bipolar Disorders (ISBD) network. It is conceivable that the professionals who are interested in the topic of online monitoring have come forward to participate in this research and are overrepresented. The same applies to the consumers; they were partly recruited in one outpatient clinic and partly in the Dutch advocacy network ‘plusminus’. That may raise a question: do the participants represent the overall target group? We think that our study sufficiently represents the target group in combining advocacy members and patients treated in an outpatient clinic. In our sample both extensive app users and users with less experience with apps were participating. This increases the chance that the findings are a balanced representation of patient experiences and opinions. Another limitation is that the FG members involved in this study were mainly highly educated, which implicates a potential higher level of digital skills and a more positive attitude towards new digital applications. A recent study found that low levels of education can be linked to meagre digital skills, low use of the internet, and anxiety for new digital developments (Vasilescu et al. 2020). It is recommended to involve more patients with lower levels of education when developing mood-monitoring devices. Although we argued that the FG mix of patients and professionals was beneficial, we cannot rule out the possibility that other participants influence the FG members’ opinions. We tried to avoid this bias through an individual member check after every meeting and the individual opinions’ inventory before the discussion started. Finally, the present study is mainly a qualitative study with a relatively small number of participants, so it remains unclear whether the results are fully generalizable.

We didn't explore the possibilities of auto-tracking and returning objective measures of symptoms options by devices.

Despite these shortcomings, we believe that the findings give a relevant overview of consumers’ and professionals’ thoughts and opinions on the topic of online mood monitoring in BD.

## Conclusion and practical implications

This study demonstrates the importance of involving stakeholders when identifying the requirements of a smartphone-based mood-charting application. We found a shared consciousness of the importance of using the Life-Chart Method as a leading principle for online mood monitoring. The design of online mood monitoring devices needs to include personalization, adjustability, strict privacy, an adjustable graphic report, and a direct link to early intervention strategies.

The value specifications are included in the follow-up of this project. Consumers and professionals will also be involved in the design and operationalization phases of the development of an online monitoring app. Our findings imply that any newly created application needs the careful integration of persuasive technology to increase adherence. This thorough approach is necessary for developing a monitoring app that stimulates sufficient compliance among people with BD. In the next step of the development of online mood monitoring, we suggest that consumers and professionals shine their light on the subject of passive sensing or EBT related tasks like activity levels, or objective sleep.

## Data Availability

The datasets used and analysed during the current study are available from the corresponding author on reasonable request.

## Abbreviations

BD: Bipolar disorder
CeHRes: Centre for e-Health Research and disease management
EPD: Electronic Patients Records (Dutch)
FG: Focus Group
FGM: Focus Group Meeting(s)
KenBiS: Kenniscentrum Bipolaire Stoornissen (Dutch national knowledge institute for BD)
LCM: Life Chart Method
P&P: Paper & pencil
RPP: Relapse prevention plan
